# Asymmetric cryptosystem based on optical scanning cryptography and elliptic curve algorithm

**DOI:** 10.1038/s41598-022-11861-x

**Published:** 2022-05-11

**Authors:** Xiangyu Chang, Wei Li, Aimin Yan, Peter Wai Ming Tsang, Ting-Chung Poon

**Affiliations:** 1 College of Mathematics and Science, Shanghai Normal University, Shanghai, 200234 China; 2 Department of Electronic Engineering Hong Kong, City University of Hong Kong, Kowloon Tong, Hong Kong SAR China; 3 Bradley Department of Electrical and Computer Engineering, Virginia Tech, Blacksburg, VA 24061 USA

**Keywords:** Optics and photonics, Physics

## Abstract

We propose an asymmetric cryptosystem based on optical scanning cryptography (OSC) and elliptic curve cryptography (ECC) algorithm. In the encryption stage of OSC, an object is encrypted to cosine and sine holograms by two pupil functions calculated via ECC algorithm from sender’s biometric image, which is sender’s private key. With the ECC algorithm, these holograms are encrypted to ciphertext, which is sent to the receiver. In the stage of decryption, the encrypted holograms can be decrypted by receiver’s biometric private key which is different from the sender’s private key. The approach is an asymmetric cryptosystem which solves the problem of the management and dispatch of keys in OSC and has more security strength than the conventional OSC. The feasibility of the proposed method has been convincingly verified by numerical and experiment results.

## Introduction

Optical image encryption has attracted much attention in recent years because of its inherent capability of high parallelism and multidimensional freedoms (amplitude, phase and polarization). Since Refrégiér and Javidi first proposed the double random phase encoding (DRPE) technique^1^, researchers have introduced many extended optical encryption methods such as a series of optical transforms^2–5^, digital holography^6–8^, joint transform correlator^9–11^ and ghost imaging^12–14^, etc. Furthermore, optical scanning cryptography (OSC)^15–19^ envisioned by Poon has become a prospective technology. Different from that of other CCD-based hologram acquisition systems, it can capture the hologram of a physical object with a fast scanning mechanism along with single-pixel recording. Indeed, some encryption systems have been proposed based on OSC. Yan et al. obtained experimental results of encryption using fingerprint keys^18^. Furthermore, they first demonstrated optical cryptography of 3-D object images in an incoherent optical system with biometric keys^19^. However, like most of optical encryption systems, OSC is a symmetric cryptosystem whose encryption key and decryption key are generally identical or mutually conjugate. The key must be transmitted through another secured channel when the encrypted image is delivered. So, it is hard to make sure the security of keys management and dispatch. Qin and Peng have proposed a novel and inspirational asymmetric cryptography based on phase-truncated Fourier transform (PTFT) and DRPE^20^, but it cannot solve the problem of management and dispatch of keys. To solve these problems, the public key cryptosystem has been introduced into optical encryption.

In a public key cryptosystem, each user has a pair of keys: one published publicly (known as the public key) and another stored in a secure location (known as the private key)^21–23^. Yuan et al. have proposed an asymmetric system based on DRPE and Rivest-Shamir-Adelman (RSA)^24^, which has simultaneous transmission for an encrypted image and a double random-phase encryption key. Meng et al. have reported an asymmetric cryptosystem combining two-step phase-shifting interferometry with RSA public-key cryptography^25^. In addition to the RSA, elliptic curve cryptography (ECC) is another popular digital encryption algorithm, which was introduced by Miller^26^ and Koblitz^27^. Compared with RSA algorithm, ECC has smaller parameters with equivalent levels of security^22,23^. Specifically, ECC based on 600-bit keys has the same security level as a 21,000-bit RSA system^23^. It will take an enormous time to solve the elliptic curves discrete logarithm problem, even if the attacker uses the fastest known algorithm. Hence, ECC is more attractive for mobile communication because of the smaller key sizes and hence the more on bandwidth saving. Indeed, ECC has been introduced to optical systems. Fan et al. proposed an asymmetric cryptosystem based on two-step phase-shifting interferometry (PSI) and ECC^28^. Abd El-Latif and Niu presented a hybrid image encryption scheme^29^, which generates a key stream using cyclic elliptic curve point and chaotic system which in turn is used for encryption of data stream from the image. Liu et al. have given a cryptanalysis of Abd El-Latif’s scheme^30^, which is based on cyclic elliptic curve and chaotic system. In addition, there are many other extended ECC methods^31–33^. However, most of those methods applied ECC algorithm by complicated encoding on the image. And some methods may be invalid by only encrypting parameters of optical cryptosystems with ECC algorithm because the optical system itself is vulnerable to ciphertext-only attack (COA). In other words, attackers can recover the plaintexts from the ciphertexts without encrypting parameters. For example, OSC is a linear encryption system which can be vulnerable to COA by using phase retrieval algorithm^34,35^. In this regard, it is necessary to develop asymmetric cryptosystems to enhance the security of the symmetric cryptosystems.

In this paper, we propose an asymmetric cryptosystem based on ECC algorithm and OSC system with biometric keys. Owing to the asymmetric operation of OSC system, high security could be achieved. And the proposed method also solves the problem of the management and dispatch of keys in the optical system. In addition, it is a simple system and does not need to encode image into numbers. The feasibility of the proposed method has been convincingly verified by numerical and experiment results. Our approach can provide an extra dimension for secure encryption, one which can leverage emerging technologies for multi-wavelength transmission and imaging.

## Optical scanning cryptography (OSC)

Optical scanning holography (OSH) is a method developed by Poon and Korpel^16^ for capturing holograms of physical objects with a single pixel sensor. Being different from other hologram acquisition methods that utilize digital cameras as the hologram recording devices, OSH is not restricted in the field of vision and the size of the hologram. Apart from hologram capturing, OSH can also be applied in optical encryption. In this section, we will give a brief introduction about optical scanning cryptography (OSC), an integration of OSH and encryption, as detailed description has been given in Ref.^16^. A 2-D array of data or function (e.g., a hologram) is denoted by a symbol in bold. For example, a 2-D array is represented by symbol ${\varvec{A}}$, and an entry at the ${y}^{th}$ row and the ${x}^{th}$ column is denoted as $A\left(x,y\right)$.

**Figure 1:**
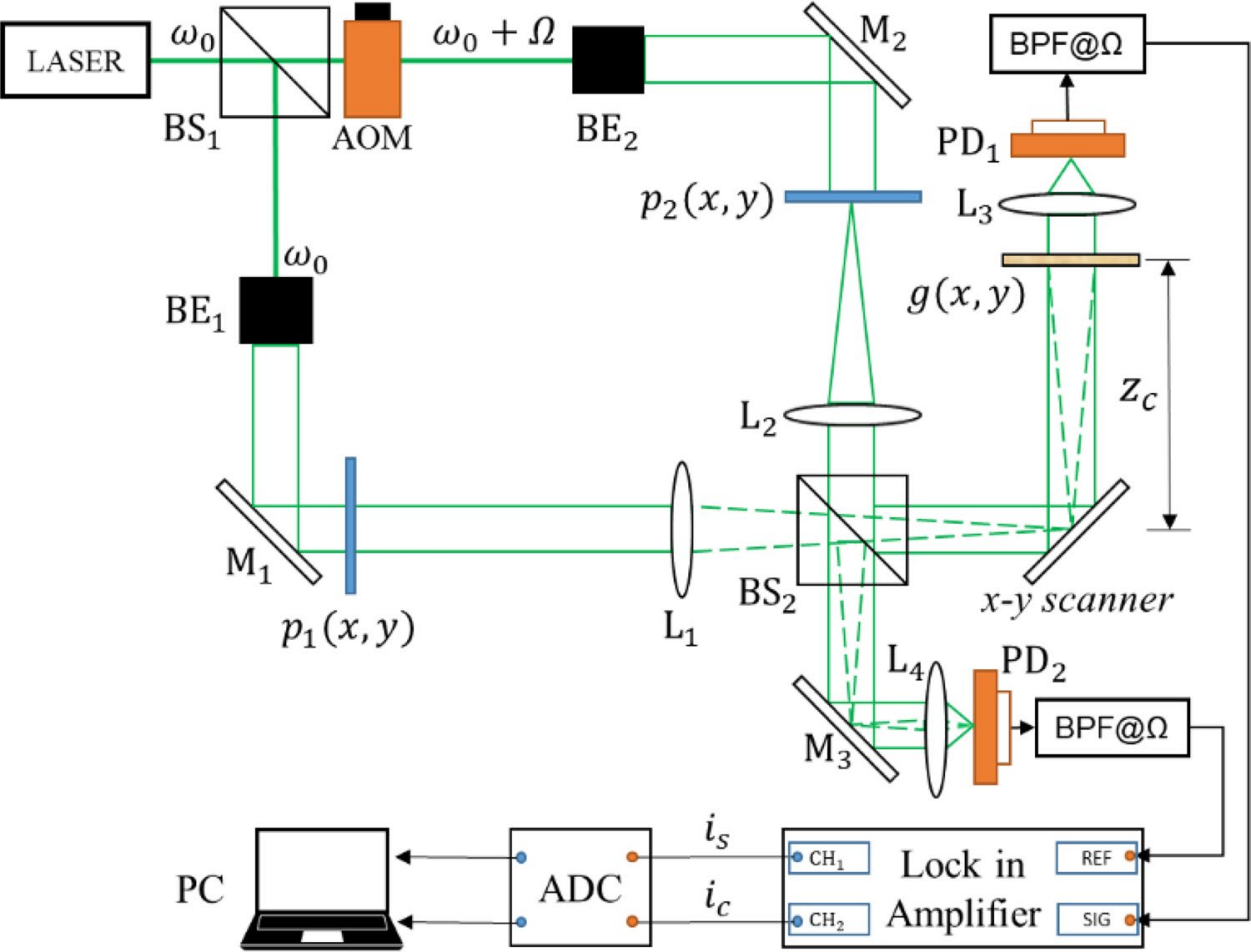
Architecture of the optical scanning cryptosystem. BS_1_ and BS_2_: beam splitters; AOM: acousto-optical modulator; BE_1_ and BE_2_: beam expanders; M_1_, M_2_ and M_3_: silver mirrors; L_1_ and L_2_: Fourier lens; L_1_ and L_2_: light-collecting lens; PD_1_ and PD_2_: photo-detectors; BPF: band-pass filter; ADC: analog-to-digital converter; PC: personal computer.

As shown in Fig. 1, both of the encryption and decryption systems are based on the architecture of Mach–Zehnder interferometer. After beam splitter (BS_1_), the laser beam with temporal frequency ${\omega }_{0}$ has been divided into two beams, and the frequency of one of the beams becomes ${\omega }_{0}+\Omega$ by using an acousto-optic modulator (AOM) operating with frequency $\Omega$. The two beams are collimated by beam expanders, BE_1_ and BE_2_, and illuminate two pupil functions ${{\varvec{p}}}_{1}$ and ${{\varvec{p}}}_{2}$, respectively. It should be noted that these two pupil functions can be utilized to perform processing on the hologram that is acquired by the OSC system. The pair of beams emerging through the two pupils pass through Fourier lens L_1_ and L_2_, and are recombined into a scanning beam by a beam splitter (BS_2_). Subsequently, the combined beam is steered in a zigzag manner with a mirror that is driven by an *x*–*y* scanner. The combined field ${\varvec{S}}$, located at a distance ${z}_{c}$ away from the back focal plane of lens L_1_, can be given as

1 $$S\left(x,y;{z}_{c}\right)=\left[FT\left\{{p}_{1}(x,y)\right\}*h\left(x,y;{z}_{c}\right)\right]\mathrm{exp}\left(j{\omega }_{0}t\right)+\left[FT\left\{{p}_{2}(x,y)\right\}*h\left(x,y;{z}_{c}\right)\right]\mathrm{exp}\left[j\left({\omega }_{0}+\Omega \right)t\right]$$

where *FT* denotes the Fourier transform, j is the imaginary unit and symbol “$*$” is the 2-D convolution operation. $h\left(x,y;{z}_{c}\right)$ denotes the free impulse response in Fourier optics^16^. The specimen is a translucent object with intensity distribution ${\varvec{g}}$, and located at an axial distance ${z}_{c}$ away from the focal plane of lens L_1_. The scanning beam is impinged on the specimen, and at each scan point photo-detector (PD) is employed to receive all the light scattered from the object, giving an electrical signal current as output. After bandpass filtering (BPF) of the signal current, heterodyne current at frequency $\Omega$ is obtained. The heterodyne current is then processed by a lock-in amplifier to give a couple of signal currents ${i}_{c}$ and ${i}_{s}$, which represent the in-phase hologram ${{\varvec{H}}}_{{\varvec{c}}{\varvec{o}}{\varvec{s}}}$, which is also called as cosine hologram, and the quadrature hologram ${{\varvec{H}}}_{{\varvec{s}}{\varvec{i}}{\varvec{n}}}$, which is also called as sine hologram, respectively. Mathematically, a complex hologram acquired with the OSC system is given by

2 $$H\left(x,y\right)={H}_{cos}\left(x,y\right)+j{H}_{sin}\left(x,y\right)=F{T}^{-1}\left\{FT\left\{{\left|g\left(x,y\right)\right|}^{2}\right\}OT{F}_{\Omega }\left({k}_{x},{k}_{y};{z}_{c}\right)\right\}$$

where $F{T}^{-1}$ denotes the inverse Fourier transforms and ${OTF}_{\Omega }$ is the optical transfer function (OTF) of the optical scanning system and expressed by

3 $$OT{F}_{\Omega }\left({k}_{x},{k}_{y};{z}_{c}\right)={\mathrm{exp}}\left[j\frac{{z}_{c}}{2{k}_{0}}\left({k}_{x}^{2}+{k}_{y}^{2}\right)\right]\iint {p}_{1}^{\dagger}\left({x}^{{{\prime}}},{y}^{{{\prime}}}\right){p}_{2}\left({x}^{{{\prime}}}+\frac{f}{{k}_{0}}{k}_{x},{y}^{{{\prime}}}+\frac{f}{{k}_{0}}{k}_{y}\right)\mathrm{exp}\left[j\frac{{z}_{c}}{2{k}_{0}}\left({x}^{^{\prime}}{k}_{x}+{y}^{^{\prime}}{k}_{y}\right)\right]d{x}^{{{\prime}}}d{y}^{{{\prime}}}$$

where symbol “†” denotes the complex conjugation. ${k}_{0}$ is the wave number and $f$ is the efficient focal length of lens L_1_ and L_2_. ${k}_{x}$ and ${k}_{y}$ denote the spatial frequencies along the $x$ and $y$ directions, respectively. From Eq. (2), we can see that the object can be encrypted by ${OTF}_{\Omega }$ determined by pupil functions ${{\varvec{p}}}_{1}$ and ${{\varvec{p}}}_{2}$.

For decryption, we replace the object with a pinhole, $\delta (x,y)$, located ${z}_{d}$ away from the back focal plane of lens L1. After the similar processing as in the encryption stage, we can obtain the pinhole hologram ${{\varvec{H}}}_{{\varvec{p}}{\varvec{i}}{\varvec{n}}}$ expressed as

4 $${H}_{pin}\left(x,y;{z}_{d}\right)=F{T}^{-1}\left\{OT{F}_{\Omega }({k}_{x},{k}_{y};{z}_{d})\right\}$$

If the two pupils are correct in the encryption and decryption stages, the decryption image ${{\varvec{H}}}_{{\varvec{d}}{\varvec{e}}}$ is easy deduced by using the following calculation:

5 $${H}_{de}\left(x,y\right)=F{T}^{-1}\left\{FT\left\{{\left|g\left(x,y\right)\right|}^{2}\right\}OT{F}_{\Omega }\times OT{F}_{\Omega }^{\dagger}\right\}={\left|g\left(x,y\right)\right|}^{2}$$

subject to condition $OT{F}_{\Omega }({k}_{x},{k}_{y};{z}_{c})\times OT{F}_{\Omega }^{\dagger}({k}_{x},{k}_{y};{z}_{d})=1$ and for ${z}_{c}={z}_{d}$.

If the pupil functions ${{\varvec{p}}}_{1}$ and ${{\varvec{p}}}_{2}$ are derived from biometric signatures, such as fingerprints, the OSC and the captured hologram are referred as biometric encrypted optical scanning cryptography (BE-OSC), and biometric encrypted optical scanning hologram (BE-OSH), respectively.

### The proposed biometric and asymmetric cryptosystem

**Figure 2:**
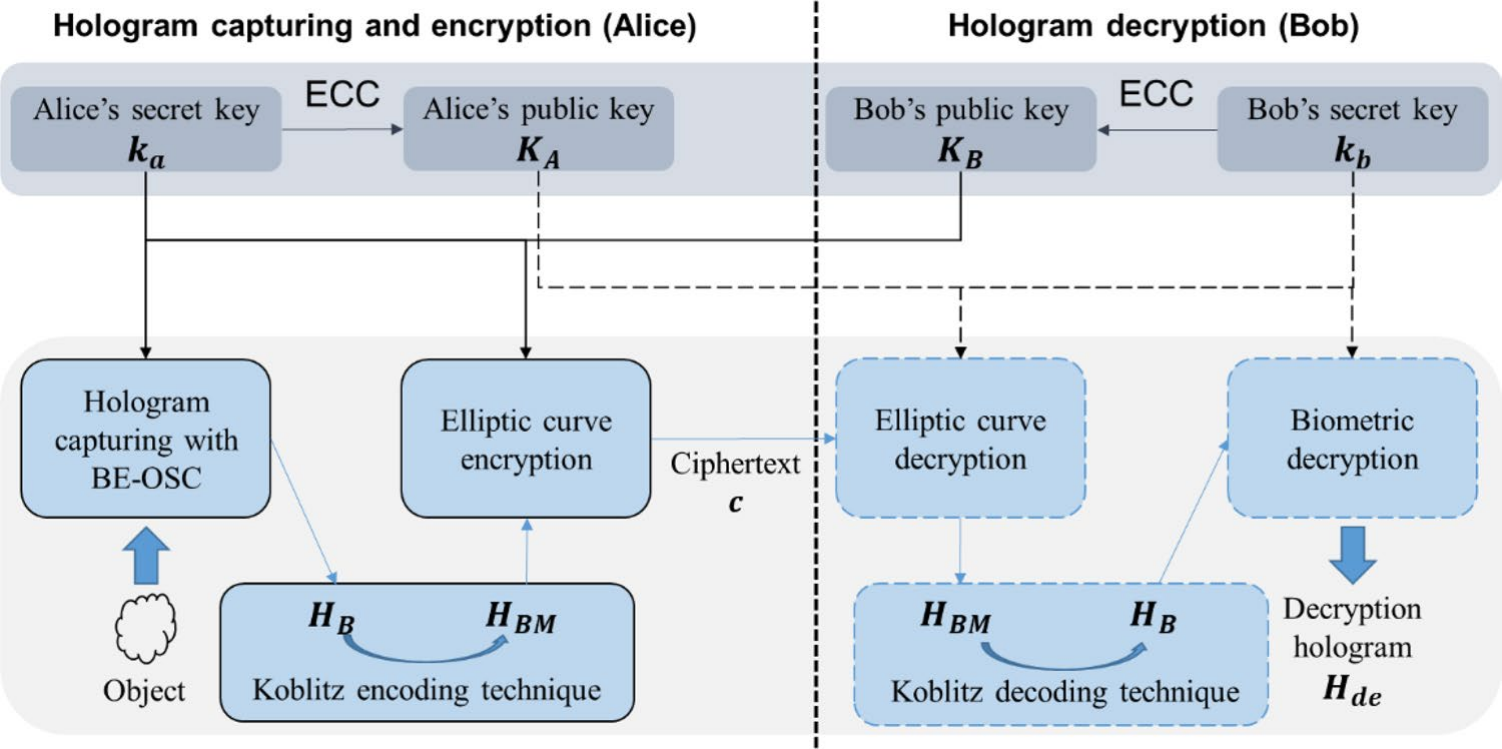
Block diagram of our proposed system.

The block diagram of our proposed method is shown in Fig. 2 and outlined as follows. To begin with, the parts on the left hand and the right hand sides of the vertical dotted line are the encryption side (operated by Alice), and the decryption side (operated by Bob), respectively. There are two shaded-shadow blocks showing different purposes. The gray blocks show the generation of secret and public keys and the blue blocks show the flow of encryption method. On the top blocks, Alice’s and Bob’s public key ${{\varvec{K}}}_{{\varvec{A}}}$ and ${{\varvec{K}}}_{{\varvec{B}}}$ are generated from their corresponding private keys ${{\varvec{k}}}_{{\varvec{a}}}$ and ${{\varvec{k}}}_{{\varvec{b}}}$ by ECC algorithm, respectively. Both sides share public keys, ${{\varvec{K}}}_{{\varvec{A}}}$ and ${{\varvec{K}}}_{{\varvec{B}}}$. We shall describe how the pair of keys are generated later. On the bottom blocks, the object is scanned by the OSC system in Fig. 1, and encrypted with the pupil functions which are derived from public key ${{\varvec{K}}}_{{\varvec{B}}}$ and private key ${{\varvec{k}}}_{{\varvec{a}}}.$ ${{\varvec{k}}}_{{\varvec{a}}}$ is a biometric image of Alice, resulting in biometric encrypted optical scanning hologram (BE-OSH) ${{\varvec{H}}}_{{\varvec{B}}}$. Subsequently, the hologram ${{\varvec{H}}}_{{\varvec{B}}}$ is embedded in ${{\varvec{H}}}_{{\varvec{B}}{\varvec{M}}}$, which is represented as elliptic curve coordinates by Koblitz encoding technique^27^. And ${{\varvec{H}}}_{{\varvec{B}}{\varvec{M}}}$ is encrypted to ciphertext ${\varvec{c}}$ by ECC using the same keys, ${{\varvec{K}}}_{{\varvec{B}}}$ and ${{\varvec{k}}}_{{\varvec{a}}}$. On the decryption side, hologram ${{\varvec{H}}}_{{\varvec{B}}{\varvec{M}}}$ is obtained from the ciphertext with public key ${{\varvec{K}}}_{{\varvec{A}}}$ and secret key ${{\varvec{k}}}_{{\varvec{b}}}$ that is only known to Bob. The biometric hologram, ${{\varvec{H}}}_{{\varvec{B}}}$, is obtained from ${{\varvec{H}}}_{{\varvec{B}}{\varvec{M}}}$ through using Koblitz decoding technique. Finally, the decryption image ${{\varvec{H}}}_{{\varvec{d}}{\varvec{e}}}$ of the object is then obtained by decrypting ${{\varvec{H}}}_{{\varvec{B}}}$ with public key ${{\varvec{K}}}_{{\varvec{A}}}$ and secret key ${{\varvec{k}}}_{{\varvec{b}}}$. In Koblitz encoding and decoding technique, plaintexts are assumed as an integer $m$. Then it is mapped to a curve point by multiplying a constant $k$ and testing all the integers $mk\le x<(m+1)k$. Obviously, $m$ can be decoded by dividing the constant $k$. In the following subsections, we shall explain the biometric encrypted OSC and the ECC in details.

#### Biometric encrypted OSC

In “Optical scanning cryptography (OSC)”, we have an overview of optical scanning cryptography. As for biometric encrypted OSC system, the pair of pupils are each replaced with a phase mask which is calculated from the user’s biometric image, such as fingerprint, iris and so on. In Fig. 2, the pair of phase masks are represented by public key ${{\varvec{K}}}_{{\varvec{B}}}$ and private key ${{\varvec{k}}}_{{\varvec{a}}}$. ${{\varvec{k}}}_{{\varvec{a}}}$ is Alice’s biometric image. The result of the scanning is biometric encrypted hologram ${{\varvec{H}}}_{{\varvec{B}}}$ and the hologram is given by

6 $${{\varvec{H}}}_{{\varvec{B}}}={{\varvec{H}}}_{{\varvec{B}}{\varvec{c}}}+j{{\varvec{H}}}_{{\varvec{B}}{\varvec{s}}}=F{T}^{-1}\left\{FT\left\{{\left|g\left(x,y\right)\right|}^{2}\right\}OT{F}_{\Omega }({k}_{x},{k}_{y};{z}_{c})\right\}$$

As such, the process will be equivalent to encrypting the holographic information with the pupil functions being the encryption keys, and hologram ${{\varvec{H}}}_{{\varvec{B}}}$ can be taken as the ciphertext of the source image ${\varvec{g}}$. From Eq. (3), we can infer that if functions ${{\varvec{p}}}_{1}$ and ${{\varvec{p}}}_{2}$ are not available to the public, the optical transfer function $OT{F}_{\Omega }({k}_{x},{k}_{y};{z}_{c})$ is unknown. Hence it is not possible to deduce the image of the specimen from biometric encrypted hologram ${{\varvec{H}}}_{{\varvec{B}}}$ through an inverse relation.

However, OSC system is vulnerable to ciphertext-only attack because it is an inherent drawback in linear optical encryption systems^34,35^. Assume that attackers only get the ciphertext, the modulus of the Fourier transform of the ciphertext can be easily obtained as follows:

7 $$\left|FT\left\{{H}_{B}(x,y)\right\}\right|=\left|FT\left\{{\left|g\left(x,y\right)\right|}^{2}\right\}\right|$$

Then the problem of recovering plaintext can be transformed into phase retrieval with a single intensity measurement. And it can be solved by using a phase retrieval algorithm, such as Gerchberg-Saxton (GS) algorithm, hybrid input–output algorithm (HIO) and so on^35^. In view of this, we have incorporated a second stage in elliptic curve cryptography (ECC) to encrypt hologram ${{\varvec{H}}}_{{\varvec{B}}}$, so as to enhance the security level of the holographic data.

#### Elliptic curve cryptography

Elliptic curve cryptography (ECC) is an asymmetric encryption method that is resistant to COA, even known-plaintext attack (KPA) which knows more assumed information than COA. As ECC has been reported in numerous literature, only a brief outline is provided for the sake of completion. ${E}_{p}$ is an elliptic curve equation over a finite field and expressed by

8 $${E}_{p}=\left\{\left(x,y\right)\in {\mathrm{R}}^{2}|{y}^{2}={x}^{3}+ax+b \left(mod p\right),4{a}^{3}+27{b}^{2}\ne 0\right\}\cup \left\{O\right\}$$

where $a$ and $b$ are two real constants, which are the parameters of the elliptic curve. Symbol “$mod$” denotes the modulo operation and $p$ is a prime number. $O$ is the identity element, a point at infinity. If a point $P(x,y)$ on addition with infinity point $O$, the result is the point itself.

9 $$P\oplus O=O\oplus P=P$$

where “$\oplus$” is point addition which is the basic operation in ECC. There are three cases in the point addition between two points, $P({x}_{1},{y}_{1})$ and $Q({x}_{2},{y}_{2})$, which add up to generate a third point $R({x}_{3},{y}_{3})$:

If ${x}_{1}\ne {x}_{2}$, the coordinate of $R$ is computed as

10 $${x}_{3}=\left\{{\lambda }^{2}-{x}_{1}-{x}_{2}\right\} mod p$$

11 $${y}_{3}=\left\{\lambda \left({x}_{1}-{x}_{3}\right)-{y}_{1}\right\} mod p$$

where

12 $$\lambda =\frac{{y}_{2}-{y}_{1}}{{x}_{2}-{x}_{1}} mod p$$

If ${x}_{1}={x}_{2}$ and ${y}_{1}={y}_{2}\ne 0$, the coordinate of $R$ is computed as

13 $${x}_{3}=\left\{{\lambda }^{2}-{2x}_{1}\right\} mod p$$

14 $${y}_{3}=\left\{\lambda \left({x}_{1}-{x}_{3}\right)-{y}_{1}\right\} mod p$$

where

15 $$\lambda =\frac{3{x}_{1}^{2}+a}{2{y}_{1}} mod p$$

If ${x}_{1}={x}_{2}$ and ${y}_{1}={y}_{2}=0$, the point will meet at infinity.

16 $$P\oplus P=O$$

If ${x}_{1}={x}_{2}$ but ${y}_{1}\ne {y}_{2}$, the third point will be a point at infinity.

17 $$P\oplus Q=O.$$

Otherwise, the point negation “$\ominus$” is expressed as

18 $$P({x}_{1},{y}_{1})\ominus Q({x}_{2},{y}_{2})=P({x}_{1},{y}_{1})\oplus Q({x}_{2},-{y}_{2})$$

In scalar multiplication “$\otimes$”, a point is multiplied with an integer $k$. The operation is realized by adding the point to itself by $k$ times. For example, if $P$ is multiplied by 3, it will be moved to a new point given by

19 $$3\otimes P=P\oplus P\oplus P$$

When parameters of elliptic curve $a,b,p$ and base point $P(x,y)$ are known, the following steps of ECC is given below.

Encryption:

- Receiver (Bob) selects a random integer ${k}_{b}$ from the interval $[1,n-1]\mathrm{as the private key}$, where $n$ is the cyclic order. The corresponding public key ${K}_{B}={k}_{b}\otimes P$ is publicized.
- The value of plaintext $m=({m}_{1},{m}_{2})$ is included in elliptic curve coordinates. And it is encrypted with a point which is obtained by scalar multiplication between Bob’s public key ${K}_{B}$ and Alice’s private key ${k}_{a}$, a random integer from the interval $[1,n-1]$. Ciphertext $c=({c}_{x},{c}_{y})$ is encrypted according to
  20 $$c=m\oplus {({k}_{a}\otimes K}_{B})$$
  Finally, the ciphertext and sender’s public key ${K}_{A}={k}_{a}\otimes P$ are sent to the receiver using the form of $\left\{{K}_{A},c\right\}$.
  Decryption:
- Receiver decrypts the ciphertext with the private key ${k}_{b}$ according to:
  21 $$m=c\ominus {({k}_{b}\otimes K}_{A})$$

### Encrypting the BE-OSC with the ECC

Next, we describe how the ECC is applied to encrypt the biometric encrypted hologram ${{\varvec{H}}}_{{\varvec{B}}}$. Without loss of generality, we assume that BE-OSC generates a square hologram of size $M\times M$. For clarity of explanation, the following terminology is defined. The sender is Alice and the receiver is Bob. ${E}_{p}(a,b)$ denotes an elliptic curve that is characterized with Eq. (8). $P(x,y)$ is the base point and ${\varvec{P}}=P\times {\varvec{I}}$ where ${\varvec{I}}$ represents a $M\times M$ unit matrix. These parameters are known to Alice and Bob. ${{\varvec{k}}}_{{\varvec{a}}}$ and ${{\varvec{k}}}_{{\varvec{b}}}$ are two $M\times M$ arrays of integers within the range $\left[1,n-1\right]$. The value of ${{\varvec{k}}}_{{\varvec{a}}}$ and ${{\varvec{k}}}_{{\varvec{b}}}$ is biometric image or randomly generated and taken to be the secret key of the user on the encryption side (i.e. Alice) and decryption side (i.e. Bob), respectively.

**Figure 3:**
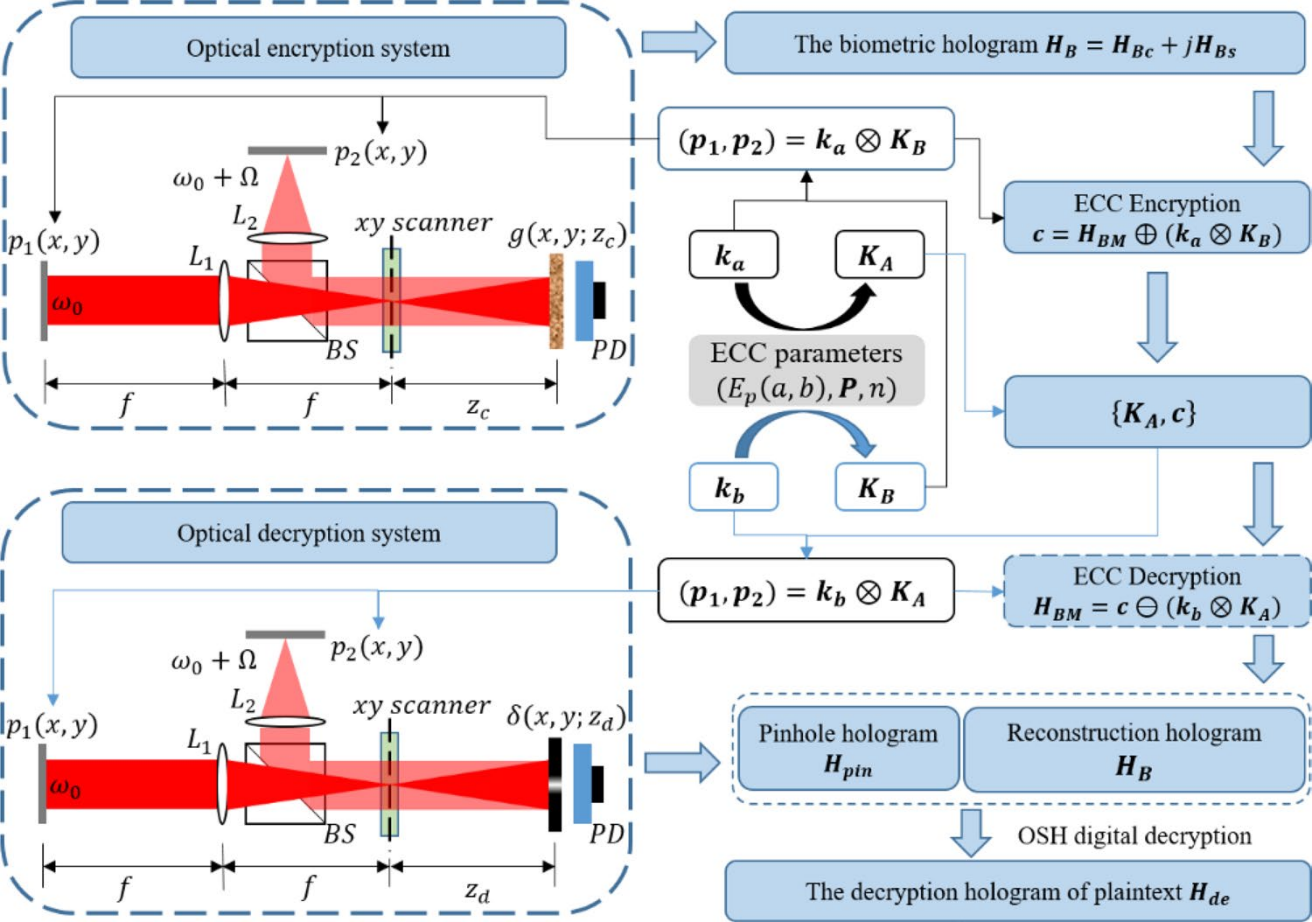
Schematic diagram of the proposed asymmetric cryptosystem.

Referring to Fig. 3, a pair of public keys, ${{\varvec{K}}}_{{\varvec{A}}}$ and ${{\varvec{K}}}_{{\varvec{B}}}$ are generated by Alice with secret key ${{\varvec{k}}}_{{\varvec{a}}}$, and Bob with secret key ${{\varvec{k}}}_{{\varvec{b}}}$, respectively, as given by

22 $${{\varvec{K}}}_{{\varvec{A}}}={{\varvec{k}}}_{{\varvec{a}}}\otimes {\varvec{P}}=({{\varvec{K}}}_{{\varvec{A}}{\varvec{x}}},{{\varvec{K}}}_{{\varvec{A}}{\varvec{y}}})$$

23 $${{\varvec{K}}}_{{\varvec{B}}}={{\varvec{k}}}_{{\varvec{b}}}\otimes {\varvec{P}}=\left({{\varvec{K}}}_{{\varvec{B}}{\varvec{x}}},{{\varvec{K}}}_{{\varvec{B}}{\varvec{y}}}\right)$$

As explain previously, the scalar multiplication in Eq. (19) is an operation to move base point $P(x,y)$ to a new position that is determined with its corresponding term in ${{\varvec{k}}}_{{\varvec{a}}}$ or ${{\varvec{k}}}_{{\varvec{b}}}$. Hence each member of ${{\varvec{K}}}_{{\varvec{A}}}$ and ${{\varvec{K}}}_{{\varvec{B}}}$ is also a point on ${E}_{p}(a,b)$, and its value is an ordered pair corresponding to the horizontal and vertical coordinates of the point.

After generation of the public keys, Bob’s public key ${{\varvec{K}}}_{{\varvec{B}}}$ is published and sent to Alice. And the pair of phase masks of the pupils that are used in the encryption stage of OSC which can be derived from ${{\varvec{K}}}_{{\varvec{B}}}$ and ${{\varvec{k}}}_{{\varvec{a}}}$ as

24 $${\left({{\varvec{p}}}_{1},{{\varvec{p}}}_{2}\right)={{\varvec{k}}}_{{\varvec{a}}}\otimes {\varvec{K}}}_{{\varvec{B}}}$$

After optical encryption, source image ${\varvec{g}}$ is encrypted to hologram ${{\varvec{H}}}_{{\varvec{B}}}={{\varvec{H}}}_{{\varvec{B}}{\varvec{c}}}+j{{\varvec{H}}}_{{\varvec{B}}{\varvec{s}}}$. As mentioned at last subsection, the source data of plaintext must belong to the elliptic curve so that ECC operators can be applied. To encrypt hologram ${{\varvec{H}}}_{{\varvec{B}}}$ obtained from BE-OSC, each pixel of the hologram is mapped to a point on the curve based on Koblitz encoding technique, resulting in hologram ${{\varvec{H}}}_{{\varvec{B}}{\varvec{M}}}=({{\varvec{H}}}_{{\varvec{B}}{\varvec{M}}{\varvec{c}}},{{\varvec{H}}}_{{\varvec{B}}{\varvec{M}}{\varvec{s}}})$. Subsequently, ${{\varvec{H}}}_{{\varvec{B}}{\varvec{M}}}$ is encrypted into a ciphertext as

25 $${{\varvec{c}}={\varvec{H}}}_{{\varvec{B}}{\varvec{M}}}\oplus \left({{\varvec{k}}}_{{\varvec{a}}}\otimes {{\varvec{K}}}_{{\varvec{B}}}\right)=\left({{\varvec{c}}}_{{\varvec{x}}},{{\varvec{c}}}_{{\varvec{y}}}\right)$$

When Bob receives $\left\{{{\varvec{K}}}_{{\varvec{A}}},{\varvec{c}}\right\}$ sent from Alice, the mapped hologram can be recovered from the ciphertext with Bob’s private key ${{\varvec{k}}}_{{\varvec{b}}}$.

26 $${{\varvec{H}}}_{{\varvec{B}}{\varvec{M}}}={\varvec{c}}\ominus \left({{\varvec{k}}}_{{\varvec{b}}}\otimes {{\varvec{K}}}_{{\varvec{A}}}\right)$$

After decryption, hologram ${{\varvec{H}}}_{{\varvec{B}}}$ can be obtained from ${{\varvec{H}}}_{{\varvec{B}}{\varvec{M}}}$ through Koblitz decoding technique. Simultaneously, two pupils are deduced by Bob’s private key ${{\varvec{k}}}_{{\varvec{b}}}$ and Alice’s public key ${{\varvec{K}}}_{{\varvec{A}}}$.

27 $${\left({{\varvec{p}}}_{1},{{\varvec{p}}}_{2}\right)={{\varvec{k}}}_{{\varvec{b}}}\otimes {\varvec{K}}}_{{\varvec{A}}}$$

Then pinhole hologram ${{\varvec{H}}}_{{\varvec{p}}{\varvec{i}}{\varvec{n}}}$ is obtained from Eq. (4). Finally, the decrypted image of the specimen ${{\varvec{H}}}_{{\varvec{d}}{\varvec{e}}}$ is decrypted from the pinhole hologram by Eq. (5).

## Experimental results

We have employed experiment to demonstrate the feasibility and effectiveness of the proposed method. The schematic of the experimental setup is shown in Fig. 1. We have adopted a 15mW He–Ne laser with λ = 632.8 nm as the coherent light source, and the heterodyne frequency (Ω/2π) is set to 25 kHz. The focal length of Lens L_1_ and L_2_ is 300 mm, and the coding distance *z*_*c*_ is 30 cm. In our experiment, we have two settings: (1) Alice’s and Bob’s private keys are their fingerprints. In reality, private keys can be any integer random matrices from interval $[1,n-1]$. (2) To obtain high-quality encrypted holograms in optical encryption system, one pupil function ${{\varvec{p}}}_{1}$ can consist of a fingerprint image $FP(x,y)$ and a positive lens with focal length ${f}_{0}$, i.e. ${p}_{1}=FP\left(x,y\right)\mathrm{exp}\left[j{k}_{0}({x}^{2}+{y}^{2})/2{f}_{0}\right]$. We use a lens with focal length of 75.6 mm to replace a random phase plate because it is a simple phase mask, albeit not random in phase distribution but easy to find in a laboratory. Another pupil is a delta function, i.e.${p}_{2}(x,y)=\delta (x,y)$. In the optical decryption system, the pinhole hologram can be obtained by putting in a pin hole as an object. These preferences are convenient and enough to demonstrate our proposed method. Based on the use of MATLAB R2016a with a personal computer, it is easy to verify the feasibility of the proposed asymmetric system.

**Figure 4:**
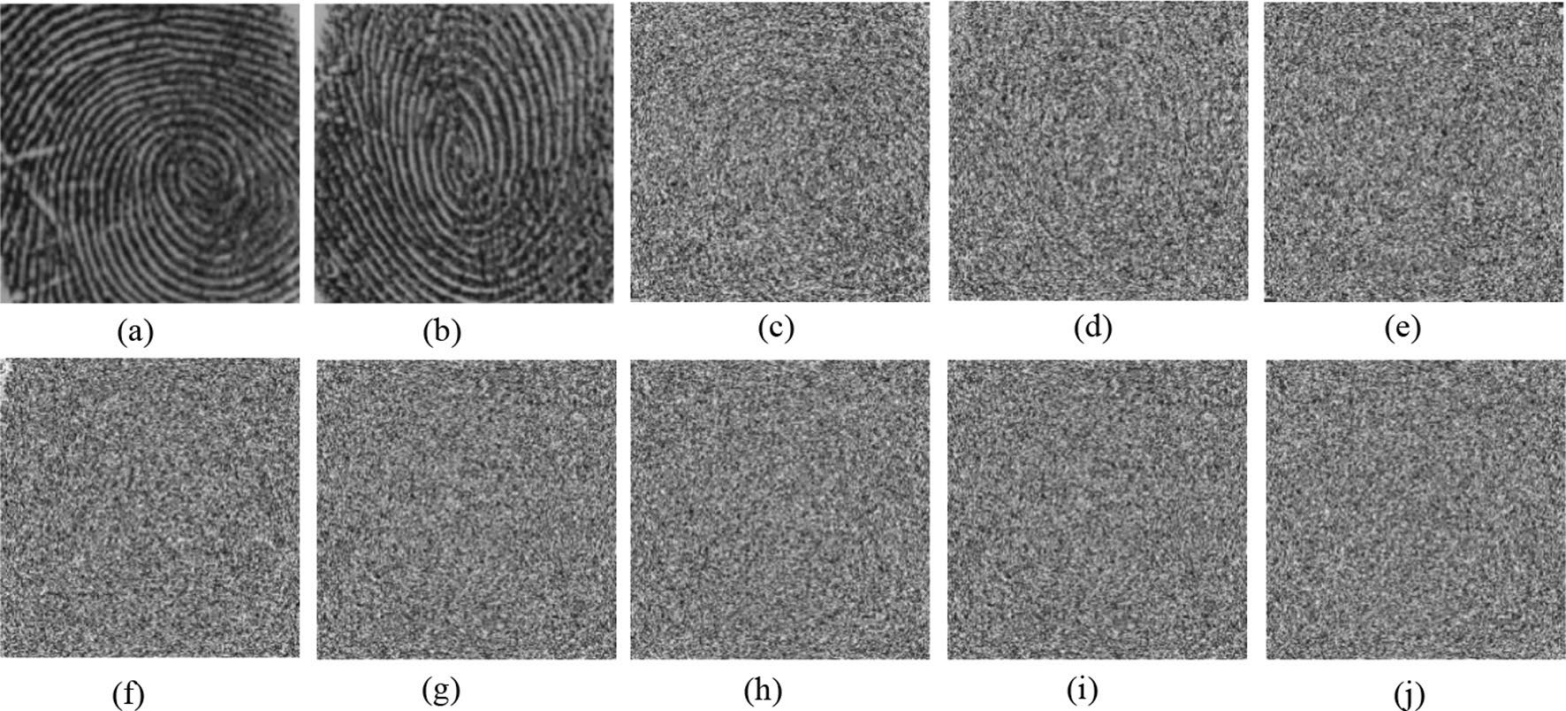
All keys in the experiment (**a**) Alice's private key ${{\varvec{k}}}_{{\varvec{a}}}$; (**b**) Bob's private key ${{\varvec{k}}}_{{\varvec{b}}}$; (**c**,**d**) are two parts of Alice's public key ${{{\varvec{K}}}_{{\varvec{A}}}=({\varvec{K}}}_{{\varvec{A}}{\varvec{x}}},{{\varvec{K}}}_{{\varvec{A}}{\varvec{y}}})$, respectively; (**e**,**f**) are two parts of Bob's public key ${{{\varvec{K}}}_{{\varvec{B}}}=({\varvec{K}}}_{{\varvec{B}}{\varvec{x}}},{{\varvec{K}}}_{{\varvec{B}}{\varvec{y}}})$, respectively; (**g**,**h**) are $({{\varvec{p}}}_{1},{{\varvec{p}}}_{2})$, generated by ${{\varvec{k}}}_{{\varvec{a}}}\otimes {{\varvec{K}}}_{{\varvec{B}}}$ in Alice’s encryption; (**i**,**j**) are $({{\varvec{p}}}_{1},{{\varvec{p}}}_{2})$, generated by ${{\varvec{k}}}_{{\varvec{b}}}\otimes {{\varvec{K}}}_{{\varvec{A}}}$ in Bob’s decryption.

**Figure 5:**
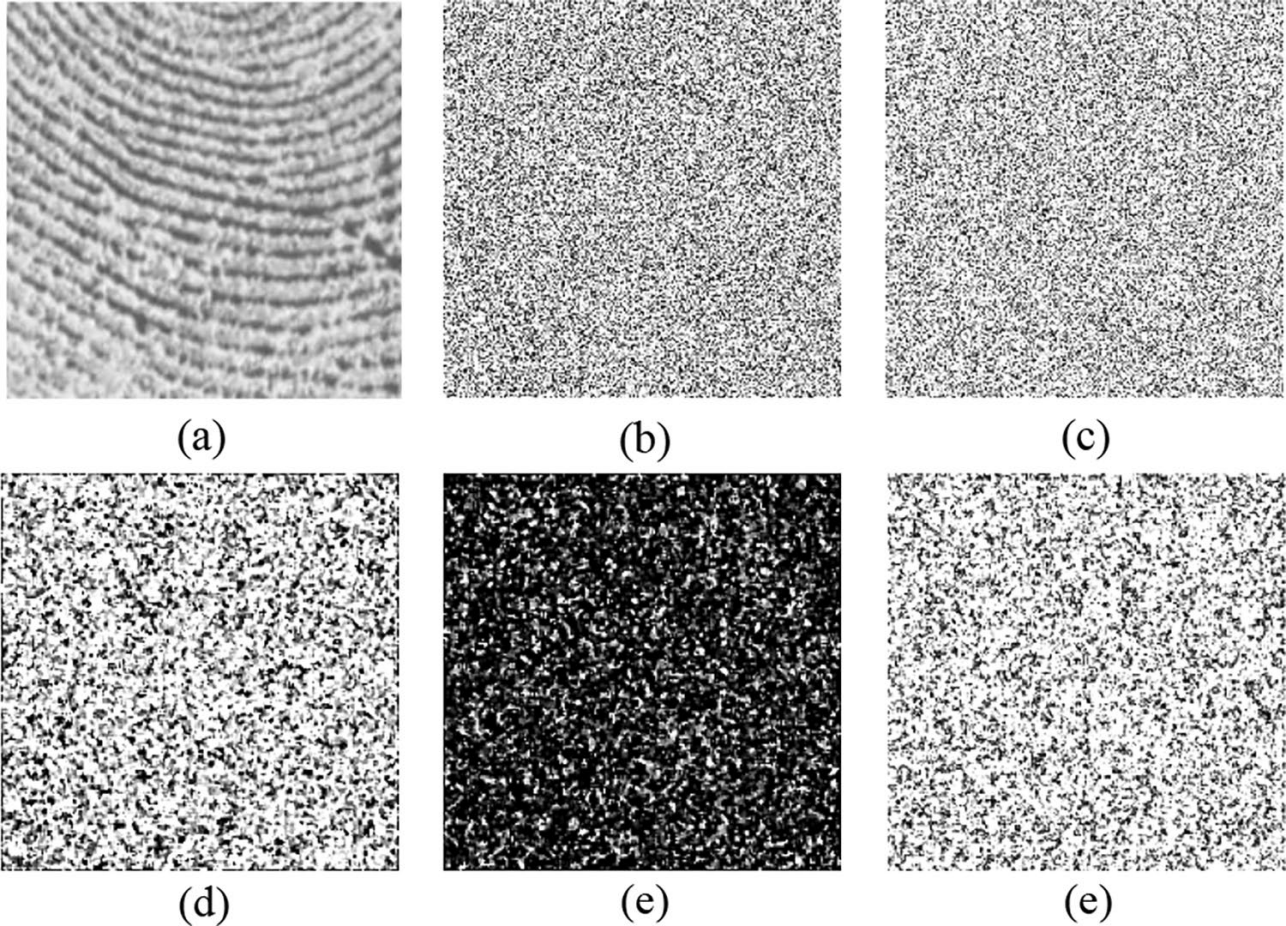
(**a**) Image to be encrypted, ${\varvec{g}}$; (**b**) final decrypted image ${{\varvec{H}}}_{{\varvec{d}}{\varvec{e}}}$; (**c**,**d**) are two parts of the mapped hologram of ‘goat’, i.e., ${{\varvec{H}}}_{{\varvec{B}}{\varvec{c}}}$ and ${{\varvec{H}}}_{{\varvec{B}}{\varvec{s}}}$ respectively; (**e**,**f**) are encrypted images, ${{\varvec{c}}}_{{\varvec{x}}}$ and ${{\varvec{c}}}_{{\varvec{y}}}$, respectively; (**g**) reconstruction cosine hologram ${{\varvec{H}}}_{{\varvec{B}}{\varvec{c}}}$; (**h**) reconstruction sine hologram ${{\varvec{H}}}_{{\varvec{B}}{\varvec{s}}}$; (**i**) and (**j**) are cosine and sine pinhole holograms ${{\varvec{H}}}_{{\varvec{B}}{\varvec{c}}}$ and ${{\varvec{H}}}_{{\varvec{B}}{\varvec{s}}}$, respectively.

**Figure 6:**
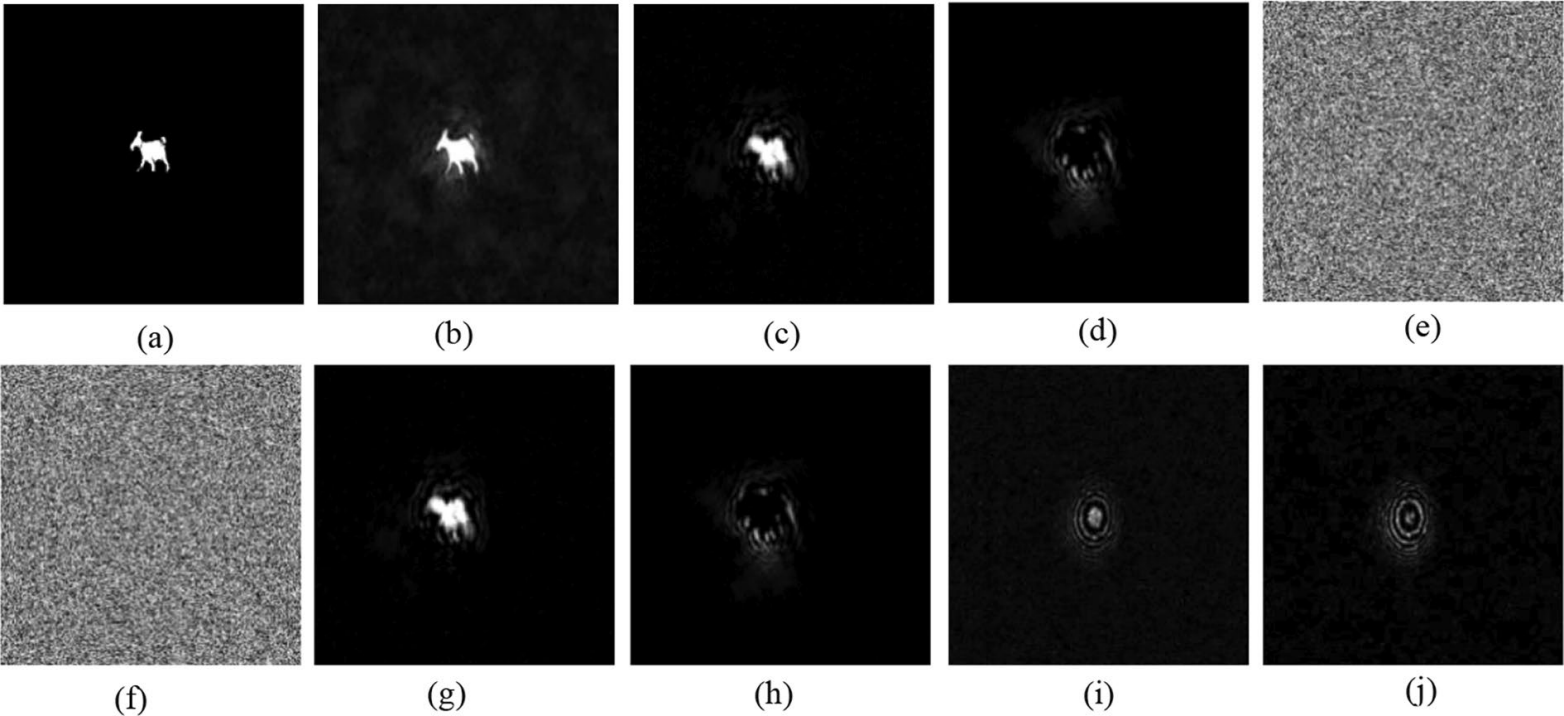
(**a**) Wrong private key ${{\varvec{w}}\_{\varvec{k}}}_{{\varvec{b}}}$; (**b**,**c**) are $({{\varvec{p}}}_{1},{{\varvec{p}}}_{2})$, generated by ${{\varvec{w}}\_{\varvec{k}}}_{{\varvec{b}}}\otimes {{\varvec{K}}}_{{\varvec{A}}}$ in decryption; (**d**,**e**) are the corresponding cosine hologram ${{\varvec{w}}\_{\varvec{H}}}_{{\varvec{B}}{\varvec{c}}}$ and sine hologram ${{\varvec{w}}\_{\varvec{H}}}_{{\varvec{B}}{\varvec{s}}}$, respectively; (**f**) decrypted image with wrong key.

To reduce the computation time, we set $a=1, b=1$ in Eq. (8) with prime number $p=29989$ and base point $P(\mathrm{29142,23400})$. Alice and Bob use their fingerprint as their private keys shown in Fig. 4a,b, respectively. Bob uses the ECC algorithm to generate Bob’s public key ${{\varvec{K}}}_{{\varvec{B}}}$ and publicizes it and ${{\varvec{K}}}_{{\varvec{B}}}$ has two parts, ${{\varvec{K}}}_{{\varvec{B}}{\varvec{x}}}$ and ${{\varvec{K}}}_{{\varvec{B}}{\varvec{y}}}$, as shown in Fig. 4e,f. When Alice wants to send the image ‘goat’ ${\varvec{g}}$, as shown in Fig. 5a, Alice needs to obtain two pupils $({{\varvec{p}}}_{1},{{\varvec{p}}}_{2})$, as shown in Fig. 4g,h, by calculating ${{\varvec{k}}}_{{\varvec{a}}}\otimes {{\varvec{K}}}_{{\varvec{B}}}$. Then, the digital holograms of plaintext are recorded by the OSC system shown in Fig. 1. The output of the OSC system is a cosine hologram ${{\varvec{H}}}_{{\varvec{B}}{\varvec{c}}}$ and a sine hologram ${{\varvec{H}}}_{{\varvec{B}}{\varvec{s}}}$, as shown in Fig. 5c,d, respectively. Next, Alice encrypts the digital holograms into the ciphertext ${\varvec{c}}$ by applying the proposed asymmetric method, which has two parts, ${{\varvec{c}}}_{{\varvec{x}}}$ and ${{\varvec{c}}}_{{\varvec{y}}}$, as shown in Fig. 5e,f, respectively. Finally, Alice sends Bob $\left\{{{\varvec{K}}}_{{\varvec{A}}},{\varvec{c}}\right\}$ where ${{\varvec{K}}}_{{\varvec{A}}}$ is Alice’s public key whose two parts are shown in Fig. 4c,d. In the decryption stage, Bob uses ${{\varvec{k}}}_{{\varvec{b}}}$ and ${{\varvec{K}}}_{{\varvec{A}}}$ to calculate the two pupils $({{\varvec{p}}}_{1},{{\varvec{p}}}_{2})$, as shown in Fig. 4i,j. Then Bob decrypts ${\varvec{c}}=({{\varvec{c}}}_{{\varvec{x}}},{{\varvec{c}}}_{{\varvec{y}}})$ and obtains the recovered cosine and sine holograms, ${{\varvec{H}}}_{{\varvec{B}}{\varvec{c}}}$ and ${{\varvec{H}}}_{{\varvec{B}}{\varvec{s}}}$, as shown in Fig. 5g,h. Simultaneously, Bob can obtain the pinhole hologram ${{\varvec{H}}}_{{\varvec{p}}{\varvec{i}}{\varvec{n}}}$, as shown in Fig. 5i,j. Finally, the decryption image ${{\varvec{H}}}_{{\varvec{d}}{\varvec{e}}}$ is successful decrypted, as shown in Fig. 5b. The proposed cryptosystem has a simple structure and requires no encoding image into numbers. And it has strong secure strength because it encrypts holograms, not parameters, in ECC stage. On the other hand, if attacker uses the wrong fingerprint shown in Fig. 6a to decrypt the system, they will get wrong results. Figure 6b,c are the two pupils $({{\varvec{p}}}_{1},{{\varvec{p}}}_{2})$ generated by ${{\varvec{w}}\_{\varvec{k}}}_{{\varvec{b}}}\otimes {{\varvec{K}}}_{{\varvec{A}}}$ in decryption. And Fig. 6d,e show the recovered cosine hologram ${{\varvec{w}}\_{\varvec{H}}}_{{\varvec{B}}{\varvec{c}}}$ and sine hologram ${{\varvec{w}}\_{\varvec{H}}}_{{\varvec{B}}{\varvec{s}}}$ with wrong key. The corresponding decrypted image is shown in Fig. 6e. We observe that the decrypted image is completely different the original image, and the contents are completely unrecognizable.

### Ethical approval

The authors confirmed that all experiments (taking fingerprints of an individual) were performed in accordance with relevant guidelines and regulations. The individual explicitly allowed the authors to use the data in the present publication.

### Informed consent

In this study, we only used fingerprints, not involving other human participants. The fingerprint used in this study is taken from Aimin Yan. Aimin Yan performed the optical experiments in optical laboratory and provided informed consent for the same.

## Further analysis and discussion

**Figure 7:**
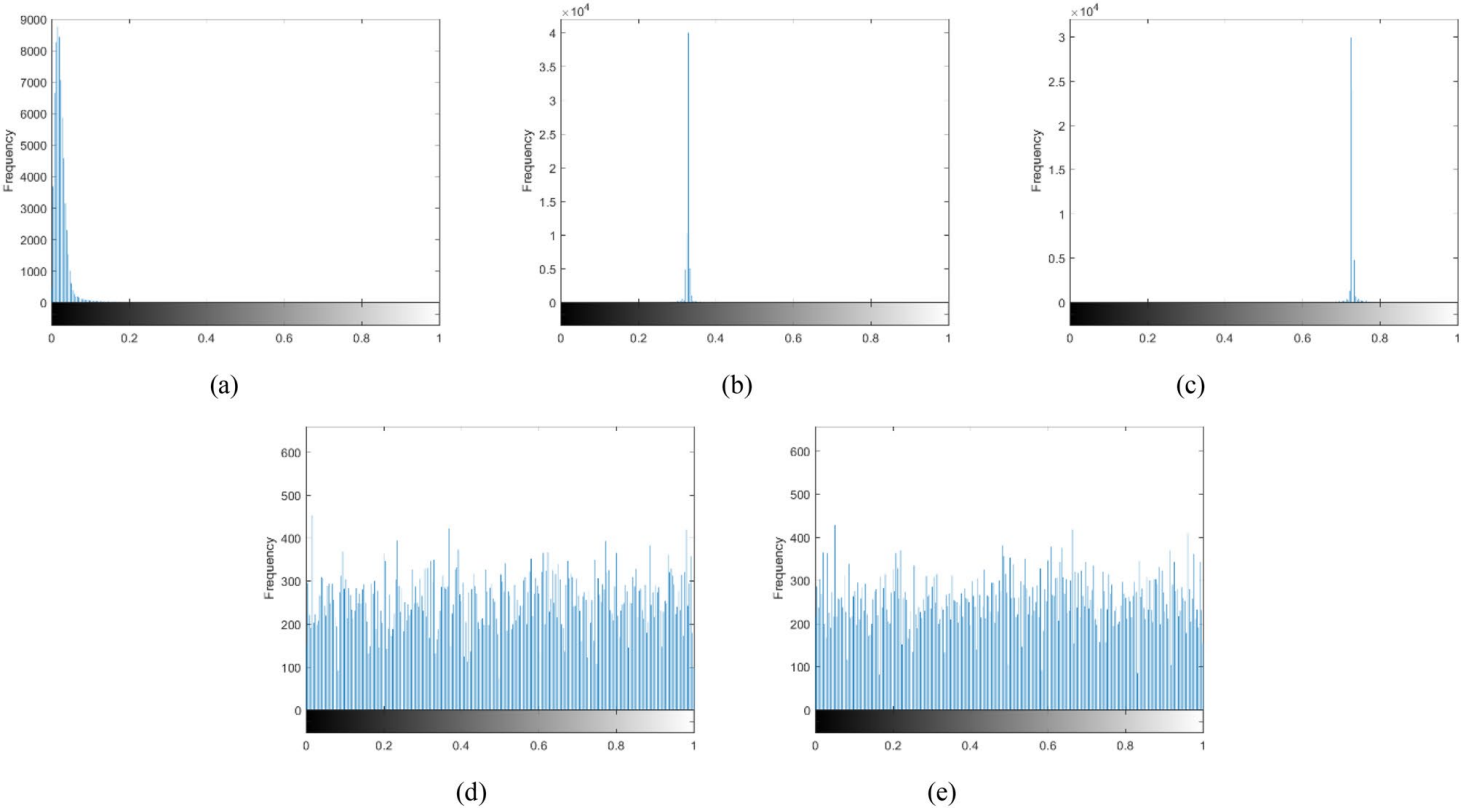
Histogram of (**a**) plaintext, (**b**) cosine hologram, (**c**) sine hologram, (**d**) ${{\varvec{c}}}_{{\varvec{x}}}$, and (e) ${{\varvec{c}}}_{{\varvec{y}}}$.

Next, we include a further analysis of the proposed method. First, the histogram of an image plots the pixel values against its frequency of occurrence. It is an important trait for ciphertext to distribute pixel values uniformly. Histogram of plaintext and its corresponding ciphertext using the proposed method are given in Fig. 7. Most of the pixel values of the “goat” are less than 0.1 in the histogram of Fig. 7a. After optical encryption, pixel values of the cosine and sine holograms distribute around 0.3 and 0.7, as shown in Fig. 7b,c, respectively. So, it may leak out information about plaintext. However, as shown in Fig. 7d,e, histograms of ciphertext are distributed equally and hence it is hard to obtain useful information from the ciphertext. These results demonstrate the proposed method works well.

**Figure 8:**
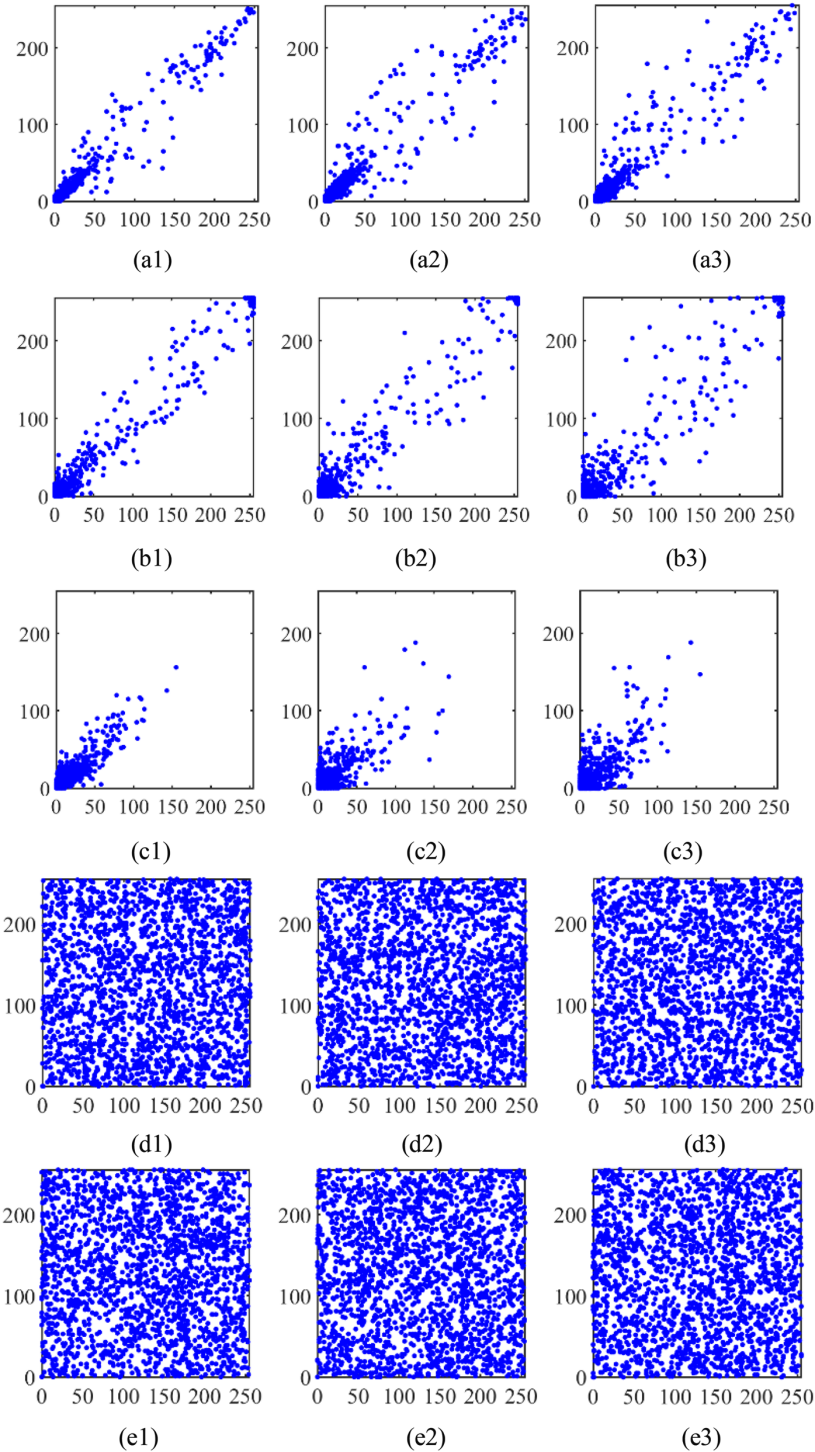
(**a1**–**a3**) The adjacent pixel distributions of plaintext in the horizontal, vertical and diagonal directions; (**b1**–**b3**) the adjacent pixel distributions of cosine hologram in the horizontal, vertical and diagonal directions; (**c1**–**c3**) the adjacent pixel distributions of sine hologram in the horizontal, vertical and diagonal directions; (**d1**–**d3**) the adjacent pixel distributions of ${{\varvec{c}}}_{{\varvec{x}}}$ in the horizontal, vertical and diagonal directions; (**e1**–**e3**) the adjacent pixel distributions of of ${{\varvec{c}}}_{{\varvec{y}}}$ in the horizontal, vertical and diagonal directions.

Second, it is necessary to analyze the correlation of adjacent pixels, which reflects the correlation of pixel values in adjacent positions. If the correlation is large, it means that the difference of gray value in the larger area of the image is small, which will affect the security of the image. Therefore, we analyze the correlation between 2000 adjacent pixels randomly selected in three directions of these images. The correlation of adjacent pixels of plaintext and its corresponding ciphertext using the proposed method are given in Fig. 8. After optical encryption, the correlation between the adjacent pixels of cosine holograms and the adjacent pixels of sine holograms are still very high, as shown in Fig. 8b1–b3 and c1–c3, respectively. However, as shown in Fig. 8d1–d3 and e1–e3, the correlation of adjacent pixels of ciphertext are very low and hence the security of ciphertext are relatively high. In addition, the correlation coefficients of these images in three directions are shown in Table 1. It is proved that the proposed method is very effective.

**Table 1:** Correlation coefficients of adjacent pixels.

Correlation coefficients	Plaintext	Cosine hologram	Sine hologram	Ciphertext	
				${{\varvec{c}}}_{{\varvec{x}}}$	${{\varvec{c}}}_{{\varvec{y}}}$
Horizontal	0.9804	0.9853	0.9278	0.0016	0.0064
Vertical	0.9637	0.9764	0.8375	–0.0042	–0.0014
Diagonal	0.9578	0.9706	0.8374	0.0169	0.0131

Third, image information entropy expresses the average amount of information in the image, which is defined by the following equation:

28 $$H\left(x\right)=-\sum_{i=0}^{255}P({x}_{i}){\mathrm{log}}_{2}P({x}_{i})$$

where *P*(*x*_*i*_) is the probability of a gray value appearing in the image. If an image is very safe, the probability of all gray values should be equal, then according to the Eq. (28), *H*(*x*) is equal to 8. And the information entropy of these images are shown in Table 2. The information entropy of ciphertext is extremely close to 8, which shows that our method is very safe.

**Table 2:** The information entropy.

Plaintext	Cosine hologram	Sine hologram	Ciphertext	
			${{\varvec{c}}}_{{\varvec{x}}}$	${{\varvec{c}}}_{{\varvec{y}}}$
1.9577	1.1599	3.3125	7.9528	7.9608

**Figure 9:**
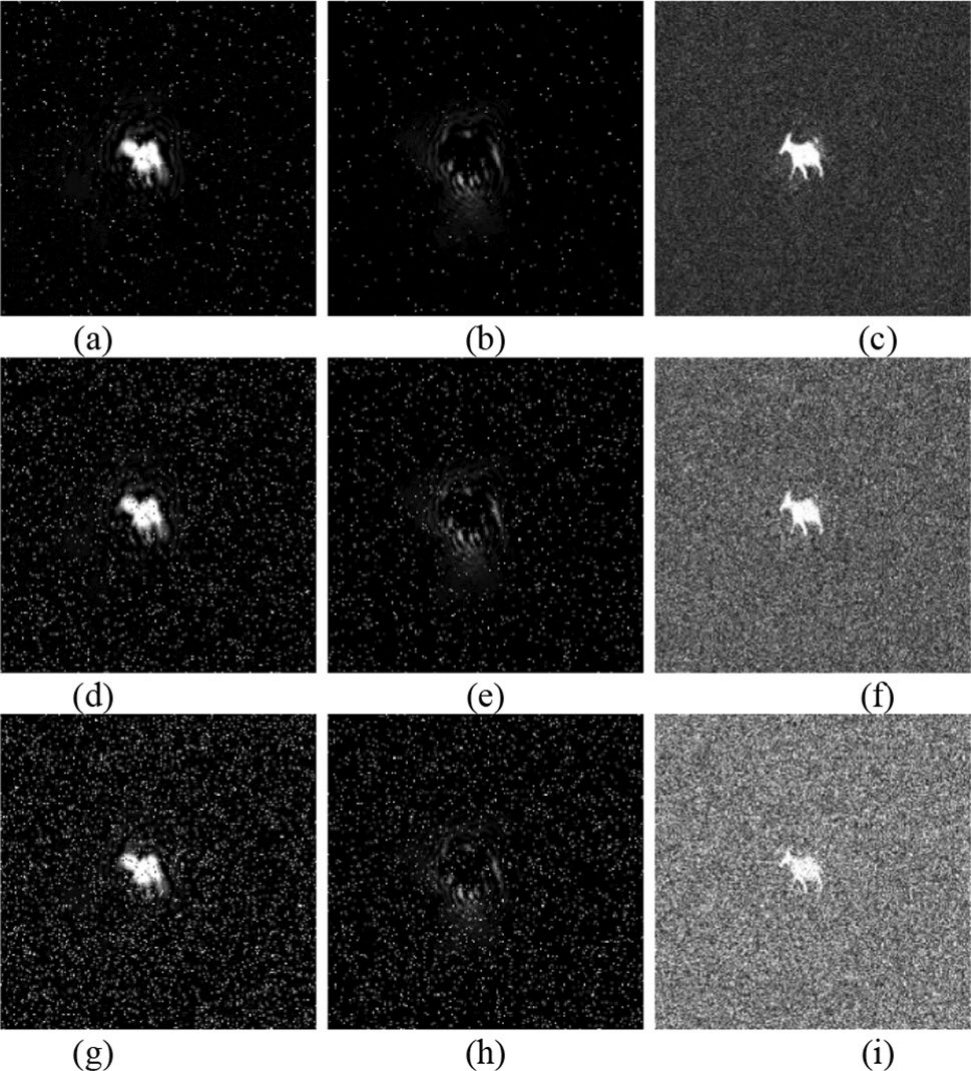
Decrypted images with salt and pepper noise (**a**–**c**) are reconstruction cosine and sine holograms and recovered image with 0.01 density, respectively; (**d**–**f**) are images with 0.05 density; (**g**–**i**) are images with 0.1 density.

**Figure 10:**
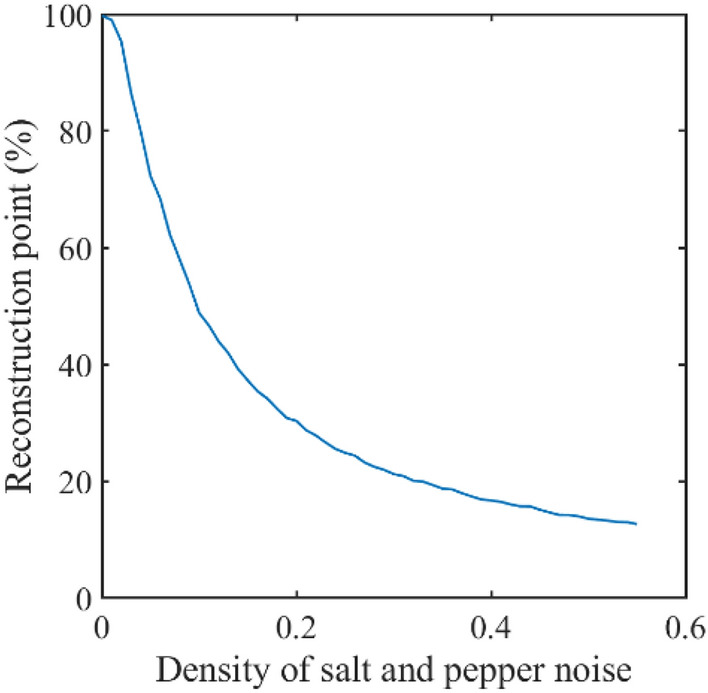
Decrypted images reconstruction rate with salt and pepper noise.

Fourth, let us consider that the ciphertext is transferred through a channel. It is possible that the receiver receives the cipher image with salt-and-pepper noise. When the receiver decrypts ciphertext with salt-and-pepper noise of 0.01 density which is the percentage of noise point that is in the total number of pixels. The reconstruction cosine and sine holograms are shown in Fig. 9a,b, respectively, and the corresponding recovered plaintext is shown in Fig. 9c. Figure 9d–f are shown with noise of 0.05 density. Finally, Fig. 9g–i are shown with noise of 0.1 density. In addition, we draw the curve between salt-and-pepper noise with different densities and image reconstruction rate, as shown in Fig. 10. These results demonstrate that the proposed cryptosystem has fairly good robustness.

**Figure 11:**
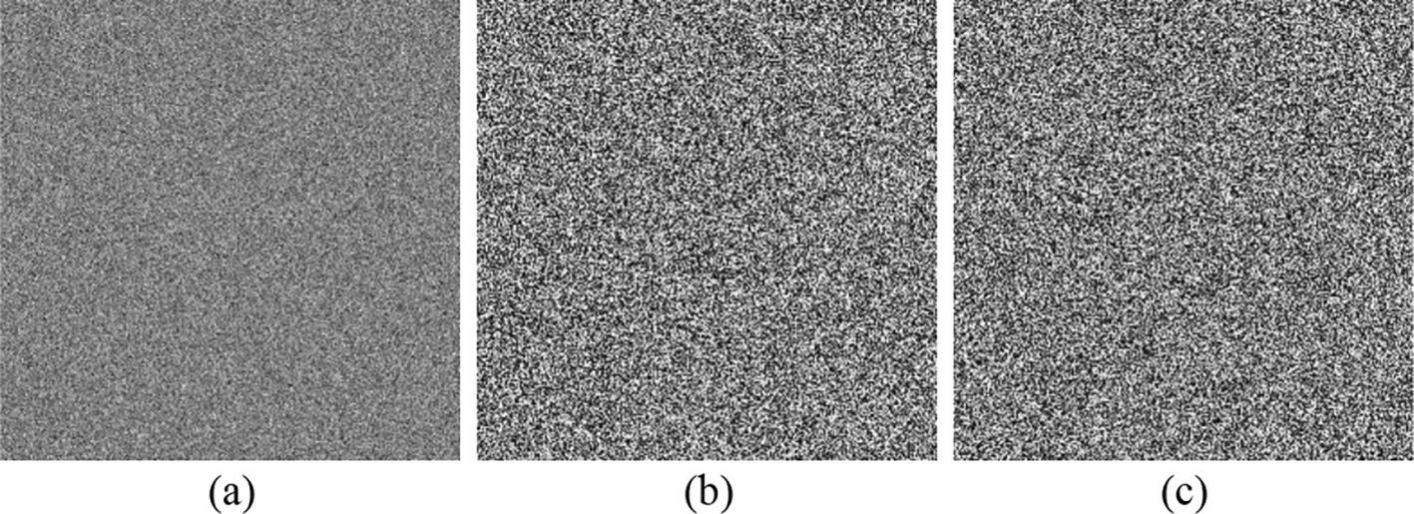
(**a**) Bob's secret key ${{\varvec{k}}}_{{\varvec{b}}}{^{\prime}}$; (**b**,**c**) are ${({{\varvec{K}}}_{{\varvec{B}}}\oplus {\varvec{k}}}_{{\varvec{b}}}{^{\prime}}\otimes {{\varvec{K}}}_{{\varvec{A}}})$, generated in Bob’s decryption.

Fifth, we should discuss known plaintext attack to further prove the security of our cryptosystem. According to the Eq. (20), ${{\varvec{K}}}_{{\varvec{B}}}=\left({{\varvec{K}}}_{{\varvec{B}}{\varvec{x}}},{{\varvec{K}}}_{{\varvec{B}}{\varvec{y}}}\right)$ as shown in Fig. 4e,f determine the cryptosystem's ability to resist known plaintext attack. If the public and fixed ${{\varvec{K}}}_{{\varvec{B}}}$ is used, it will be vulnerable to known plaintext attack, but changing the value of ${{\varvec{K}}}_{{\varvec{B}}}$ frequently will make our cryptosystem more complicated. In order to solve this problem, Bob can randomly generate a secret key ${{\varvec{k}}}_{{\varvec{b}}}\boldsymbol{^{\prime}}$ and transmit $\left\{{{\varvec{k}}}_{{\varvec{b}}^{\prime}}\otimes {\varvec{P}},{({{\varvec{K}}}_{{\varvec{B}}}\oplus {\varvec{k}}}_{{\varvec{b}}^{\prime}}\otimes {{\varvec{K}}}_{{\varvec{A}}})\right\}$ to Alice, as shown in the Fig. 11. Then Alice calculates the following equation:

29 $${{{\varvec{K}}}_{{\varvec{B}}}\oplus {\varvec{k}}}_{{\varvec{b}}}^{{\prime}}\otimes {{\varvec{K}}}_{{\varvec{A}}} \ominus {{\varvec{k}}}_{{\varvec{A}}}\otimes {{\varvec{k}}}_{{\varvec{b}}}^{{\prime}}\otimes {\varvec{P}}={{\varvec{K}}}_{{\varvec{B}}}$$

where ${{\varvec{K}}}_{{\varvec{A}}}={{\varvec{k}}}_{{\varvec{A}}}\otimes {\varvec{P}}$. Therefore, ${{\varvec{K}}}_{{\varvec{B}}}$ will be hidden and our cryptosystem can resist known plaintext attack.

## Conclusion

We have proposed a novel asymmetric cryptosystem that combines optical scanning cryptography (OSC) with the elliptic curve public-key cryptographic algorithm. Simulation and experimental results have verified the feasibility of this method. The proposed method has the following advantages. First, the system realizes asymmetric encryption because the ways to obtain the encryption and decryption keys are different and the dispatch of keys does not need to be considered. Second, the cosine and sine holograms are nonlinearly encrypted simultaneously, so its security level is better than the conventional OSC system. Third, the overall system has good robustness and its ciphertext will not leak information of the plaintext. The proposed asymmetric cryptosystem for enhancing the security of OSC is also applicable to other acquired digital holograms from conventional digital holography for optical imaging encryption.

## Data Availability

The datasets generated during and/or analyzed during the current study are available from the corresponding author on reasonable request.

## References

[1] Refregier P, Javidi B (1995). Optical image encryption based on input plane and fourier plane random encoding. Opt. Lett..

[2] Situ G, Zhang J (2004). Double random-phase encoding in the Fresnel domain. Opt. Lett..

[3] Li H, Wang Y (2008). Double-image encryption based on iterative gyrator transform. Opt. Commun..

[4] Sui L, Xin M, Tian A (2013). Multiple-image encryption based on phase mask multiplexing in fractional Fourier transform domain. Opt. Lett..

[5] Singh P, Yadav AK, Singh K (2017). Phase image encryption in the fractional Hartley domain using Arnold transform and singular value decomposition. Opt. Lasers Eng..

[6] Javidi B, Nomura T (2000). Securing information by use of digital holography. Opt. Lett..

[7] Chen L, Zhao D (2007). Color information processing (coding and synthesis) with fractional Fourier transforms and digital holography. Opt. Express.

[8] Rajput SK, Matoba O (2017). Optical voice encryption based on digital holography. Opt. Lett..

[9] Nomura T, Javidi B (2000). Optical encryption using a joint transform correlator architecture. Opt. Eng..

[10] Zea AV, Ramirez JFB, Torroba R (2016). Three-dimensional joint transform correlator cryptosystem. Opt. Lett..

[11] Vilardy JM, Millán MS, Pérez-Cabré E (2017). Nonlinear image encryption using a fully phase nonzero-order joint transform correlator in the Gyrator domain. Opt. Lasers Eng..

[12] Clemente P, Durán V, Tajahuerce E, Lancis J (2010). Optical encryption based on computational ghost imaging. Opt. Lett..

[13] Tanha M, Kheradmand R, Ahmadikandjani S (2012). Gray-scale and color optical encryption based on computational ghost imaging. Appl. Phys. Lett..

[14] Wang F, Wang H, Wang H, Li G, Situ G (2019). Learning from simulation: An end-to-end deep-learning approach for computational ghost imaging. Opt. Express.

[15] Poon T-C, Kim T, Doh K (2003). Optical scanning cryptography for secure wireless transmission. Appl. Opt..

[16] T.-C. Poon. Optical scanning holography with MATLAB. **21**, New York, NY: Springer, 2007. 10.1007/978-0-387-68851-0

[17] Yan A, Sun J, Hu Z, Zhang J, Liu L (2015). Novel optical scanning cryptography using Fresnel telescope imaging. Opt. Express.

[18] Yan A, Poon T-C, Hu Z, Zhang J (2016). Optical image encryption using optical scanning and fingerprint keys. J. Mod. Opt..

[19] Yan A, Wei Y, Hu Z, Zhang J, Tsang PWM, Poon T-C (2017). Optical cryptography with biometrics for multi-depth objects. Sci. Rep..

[20] Qin W, Peng X (2010). Asymmetric cryptosystem based on phase-Truncated Fourier Transforms. Opt. Lett..

[21] Diffie W, Hellman M (1976). New directions in cryptography. IEEE Trans. Inf. Theory.

[22] Vanstone S (2003). Next generation security for wireless: Elliptic curve cryptography. Comput. Secur..

[23] Hankerson D, Menezes A (2011). Elliptic curve cryptography.

[24] Yuan S, Zhou X, Li DH, Zhou DF (2007). Simultaneous transmission for an encrypted image and a double random-phase encryption key. Appl. Opt..

[25] Meng XF, Peng X, Cai LZ, Li AM, Gao Z, Wang YR (2009). Cryptosystem based on two-step phase-shifting interferometry and the RSA public-key encryption algorithm. J. Opt. A Pure Appl. Opt..

[26] Miller, V. S. Use of elliptic curves in cryptography. Conference on the theory and application of cryptographic techniques. Springer, Berlin, Heidelberg. 10.1007/3-540-39799-X_31 (1985).

[27] Koblitz N (1987). Elliptic curve cryptosystems. Math. Comput..

[28] Fan D, Meng X, Wang Y, Yang X, Peng X, He W, Chen H (2013). Asymmetric cryptosystem and software design based on two-step phase-shifting interferometry and elliptic curve algorithm. Opt. Commun..

[29] Abd El-Latif AA, Niu X (2013). A hybrid chaotic system and cyclic elliptic curve for image encryption. AEU-Int. J. Electron. Commun..

[30] Liu H, Liu Y (2014). Cryptanalyzing an image encryption scheme based on hybrid chaotic system and cyclic elliptic curve. Opt. Laser Technol..

[31] Tawalbeh L, Mowafi M, Aljoby W (2013). Use of elliptic curve cryptography for multimedia encryption. IET Inf. Secur..

[32] Laiphrakpam DS, Khumanthem MS (2017). Medical image encryption based on improved ElGamal encryption technique. Optik.

[33] Khoirom MS, Laiphrakpam DS, Themrichon T (2018). Cryptanalysis of multimedia encryption using elliptic curve cryptography. Optik.

[34] Li G, Yang W, Li D, Situ G (2017). Cyphertext-only attack on the double random-phase encryption: Experimental demonstration. Opt. Express.

[35] Chang X, Yan A, Zhang H (2020). Ciphertext-only attack on optical scanning cryptography. Opt. Lasers Eng..

